# Genetic signatures of adaptation revealed from transcriptome sequencing of Arctic and red foxes

**DOI:** 10.1186/s12864-015-1724-9

**Published:** 2015-08-07

**Authors:** Vikas Kumar, Verena E. Kutschera, Maria A. Nilsson, Axel Janke

**Affiliations:** Senckenberg Biodiversity and Climate Research Center, Senckenberg Gesellschaft für Naturforschung, Senckenberganlage 25, D-60325 Frankfurt am Main, Germany; Department of Evolutionary Biology, Evolutionary Biology Centre, Uppsala University, Norbyvägen 18D, SE-75236 Uppsala, Sweden; Evolution & Diversity, Goethe University Frankfurt, Institute for Ecology, Biologicum, Max-von-Laue-Str.13, D-60439 Frankfurt am Main, Germany

**Keywords:** Arctic fox, *Vulpes lagopus*, Red fox, *Vulpes vulpes*, Transcriptome, Positive selection, Arctic adaptation

## Abstract

**Background:**

The genus *Vulpes* (true foxes) comprises numerous species that inhabit a wide range of habitats and climatic conditions, including one species, the Arctic fox (*Vulpes lagopus*) which is adapted to the arctic region. A close relative to the Arctic fox, the red fox (*Vulpes vulpes*), occurs in subarctic to subtropical habitats. To study the genetic basis of their adaptations to different environments, transcriptome sequences from two Arctic foxes and one red fox individual were generated and analyzed for signatures of positive selection. In addition, the data allowed for a phylogenetic analysis and divergence time estimate between the two fox species.

**Results:**

The *de novo* assembly of reads resulted in more than 160,000 contigs/transcripts per individual. Approximately 17,000 homologous genes were identified using human and the non-redundant databases. Positive selection analyses revealed several genes involved in various metabolic and molecular processes such as energy metabolism, cardiac gene regulation, apoptosis and blood coagulation to be under positive selection in foxes. Branch site tests identified four genes to be under positive selection in the Arctic fox transcriptome, two of which are fat metabolism genes. In the red fox transcriptome eight genes are under positive selection, including molecular process genes, notably genes involved in ATP metabolism. Analysis of the three transcriptomes and five Sanger re-sequenced genes in additional individuals identified a lower genetic variability within Arctic foxes compared to red foxes, which is consistent with distribution range differences and demographic responses to past climatic fluctuations. A phylogenomic analysis estimated that the Arctic and red fox lineages diverged about three million years ago.

**Conclusions:**

Transcriptome data are an economic way to generate genomic resources for evolutionary studies. Despite not representing an entire genome, this transcriptome analysis identified numerous genes that are relevant to arctic adaptation in foxes. Similar to polar bears, fat metabolism seems to play a central role in adaptation of Arctic foxes to the cold climate, as has been identified in the polar bear, another arctic specialist.

**Electronic supplementary material:**

The online version of this article (doi:10.1186/s12864-015-1724-9) contains supplementary material, which is available to authorized users.

## Background

The genus *Vulpes* (foxes) comprises of twelve extant species that inhabit diverse habitats, including subtropics, deserts, temperate climatic zones as well as the Arctic [1]. The Arctic fox (*Vulpes lagopus*) is the only fox species occurring in the Arctic and is adapted to its extreme climatic conditions. Arctic foxes have to cope with extremely cold and dark winters without being able to hibernate, followed by short summer seasons to reproduce. Several evolutionary adaptations such as insulating and camouflage colored fur, a compact body, and reduced metabolism during starvation or extremely cold weather enable Arctic foxes to survive under these harsh conditions [2].

A closely related species of the Arctic fox is the red fox (*Vulpes vulpes*). The two species diverged from their most recent common ancestor approximately 2.9 million years ago (Ma) [3]. In contrast to the Arctic fox, the red fox occupies a wide range of predominantly temperate habitats, including forests, farmland, urban areas, but also deserts and tundra. The generalist habitat requirements of the red fox and its high mobility are drivers for the evolutionary success of this species, which has the widest geographical distribution of any carnivore [4]. The closely related Arctic and red foxes differ in their morphological and ecological adaptations to their respective habitats, rendering them an interesting species pair for studying the genetic basis of adaptation.

The adaptations of the two fox species are expected to have left signatures in the protein-coding genes. A widely used method to identify candidate genes that are under positive selection is calculating the dN-dS ratio (ω). This is the ratio of the number of non-synonymous substitutions per non-synonymous site (dN) and the number of synonymous substitution per synonymous site (dS). An ω > 1 indicates positive selection, an ω < 1 indicates negative (purifying) selection, while an ω ≈ 1 implies neutral evolution [5]. Different methods have been developed to calculate the ω values ranging from pair wise comparisons [6] to phylogeny based methods [7, 8]. Phylogeny based methods have the advantage to implement maximum-likelihood and evolutionary models to identify positive selection on specific lineages, rather than averaging over sequences between species pairs [7, 8]. The model-based branch site test “test 2” implemented in the PAML package [9] uses a phylogenetic tree to identify branch (lineage) specific selection at individual codons with an empirical Bayes approach. “Test 2” has been shown to be robust and yield fewer false positives compared to other methods [7, 8, 10]. The pairwise analyses and branch site test may not necessarily yield overlapping results but complement each other, because by averaging over the entire protein-coding sequence the pairwise analysis is more conservative than the branch site tests.

Recent studies analyzing whole genomes of polar and brown bears, an Arctic/non-Arctic species pair with comparable habitat ranges and adaptations to that of Arctic and red foxes, identified a number of candidate genes involved in fatty-acid metabolism, cardiovascular functions, and fur color to be under positive selection [11]. From the two fox species, whole genome sequences are not available, to investigate if a similar set of genes is under selection, to study possible convergent evolution between foxes as reported in other mammals [12, 13]. However transcriptome sequencing can economically produce large amounts of protein-coding sequence data for evolutionary analysis [14–17]. Another benefit is that *de novo* sequence assembly of transcriptome data is less complex than for whole genomes. Therefore, the aim of this study is to produce transcriptomes of Arctic and red foxes for identifying candidate genes and metabolic pathways that might be involved in arctic adaptation and to estimate phylogenomic based divergence times. Genes under positive selection will be chosen from transcripts with high abundance in terms of Fragments Per Kilobase per transcript per Million mapped reads (FPKM) and transcriptomes from two fox individuals will be used to verify the sequence data. Compared with positive selection analyses of data from a single individual, this stringent approach reduces sequencing and assembly errors that can introduce a bias in positive selection analysis [18]. In addition we compare sequence variation between the two fox species and the dog (*Canis lupus*), and we provide a resource of potential variable microsatellites, which will be useful in future population studies.

## Results

### Transcriptome sequencing and functional annotation of Arctic and red fox genes

Three transcriptome libraries were generated from pooled RNA-extracts from different tissues (liver, brain and kidney) of two Arctic fox individuals (Arctic fox1, Arctic fox2) and one red fox (Red fox). The sequencing produced more than 300 million reads with an average read length of 94 base pairs, bp (Additional file 1: Table S1). Standard quality filtering of raw reads to remove adapters, low quality reads (Q < 20) and using minimum read length of 30 bp resulted in the removal of on average 11.8 % reads (Additional file 1: Table S1). Statistics of the *de novo* assembly of each of the three fox individuals are shown in Table 1. Filtering steps that included abundance filtration by selecting transcripts with FPKM ≥ 1 and grouping similar transcripts on the basis of sequence similarity (clustering) discarded on an average 46 % transcripts for each of the three fox samples (Additional file 2: Table S2). In addition we checked FPKM of orthologous genes between the Arctic and red foxes by mapping the reads of one fox species to itself and to another fox species assembly, indicating that the orthologous genes expression are positive correlated (correlation of 0.90) (Additional file 3: Figure S1) confirming the assembly quality and a similar expression pattern.

**Table 1:** *De novo a*ssembly statistics for the three fox individuals

Assembly statistics	Arctic fox1 (VlagF01)	Arctic fox2 (VlagF02)	Red fox (VvulF01)	Pooled Arctic foxes (VlagF01 + VlagF02)
Total number of transcripts	139910	187219	156656	258600
Total assembled nucleotide bases	141142680	216201659	156970631	282473292
N50 contig length	2085	2700	2128	2774
Mean contig length	1008.8	1154.8	1002.0	1092.3
Median contig length	443	423	421	369

In addition the two Arctic fox individuals were used to generate a pooled assembly. Lab ID is shown in brackets

**Fig. 1:**
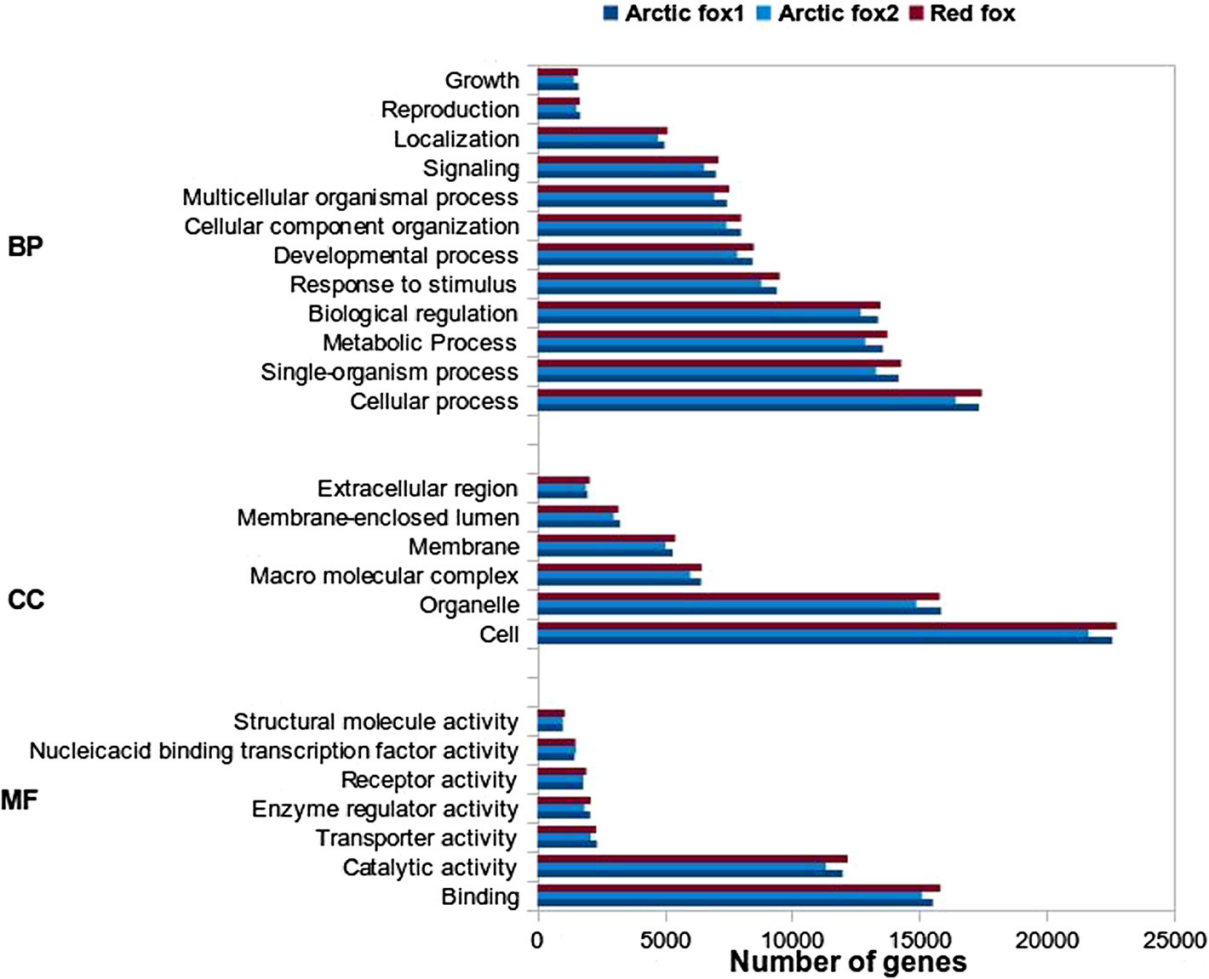
Gene ontology category distribution of three fox individuals transcripts. Gene ontology categories include Biological process (BP), Molecular function (MF) and Cellular component (CC)

Functional annotation of all assembled and identified expressed transcripts using Blast2GO resulted in 29,999, 28,956 and 30,298 transcripts that have at least one gene ontology (GO) term, for Arctic fox1, Arctic fox2 and red fox respectively corresponding to an average of approximately 35 % of each of the final transcripts. The remaining unannotated transcripts might represent non-coding RNAs, 3′ or 5′ untranslated regions, or fox-specific transcripts. GO annotation based on the GOslim database divided the functional annotations into the three main ontology components: “biological process”, “molecular function” and “cellular component”. The distribution of the number of genes in the three different GO subcategories for each species is shown in Fig. 1. The similar distribution in three GO categories of the two Arctic fox and red fox genes indicates that no major bias was introduced during the generation of the cDNA libraries. In the category “biological process” the largest number of genes was assigned to cellular processes, single organism processes and metabolic processes. In the category “molecular function”, binding and catalytic activity received most transcripts, and in the category “cellular component”, most genes were assigned to the “cell and organelle” category.

**Fig. 2:**
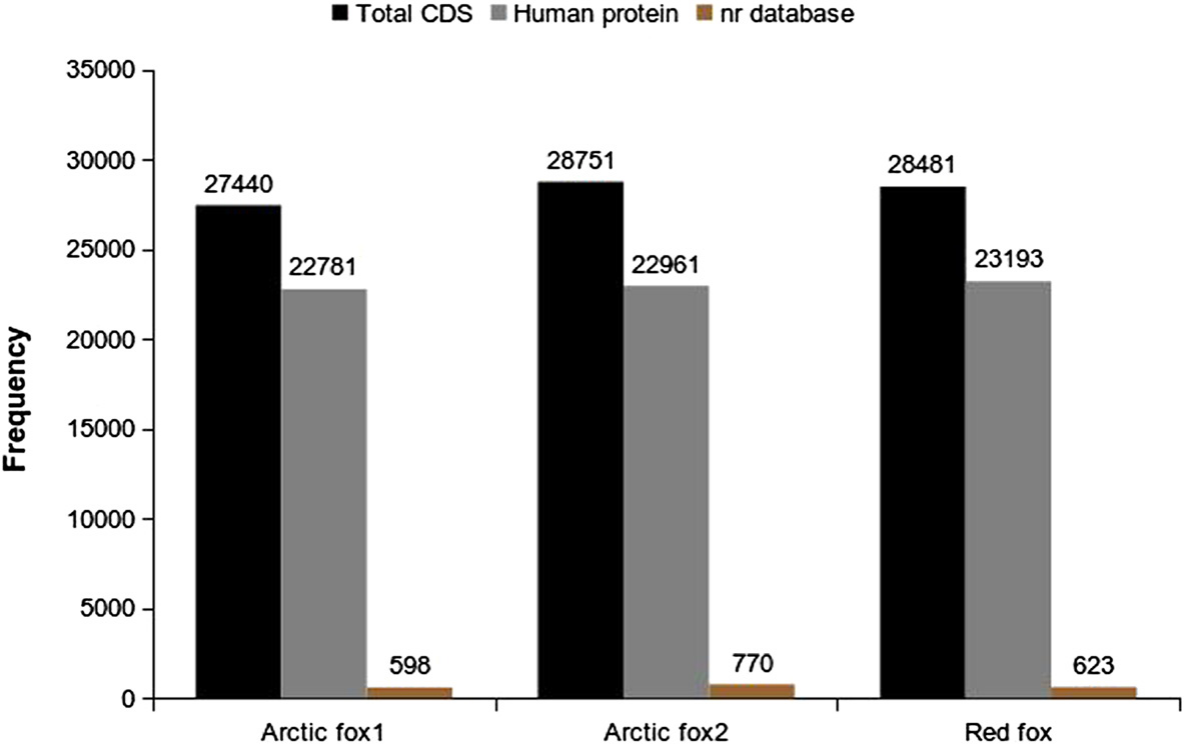
Fox coding sequence hits to the human and the non-redundant databases. The number of annotated genes for each of the three fox individuals. The black bars indicate the total number of predicted CDS, the grey bars the number of predicted CDS that have a homologue in the human protein database. The brown bars indicates the unannoated CDS from human protein database identified in non-redundant database

After coding sequence (CDS) prediction of the transcripts, only CDS longer than 300 nucleotides (nt) were annotated to avoid spurious hits. All CDS were screened against the human protein database using BLAST and 82 % of the fox genes could be annotated (Fig. 2). The remaining, un-annotated genes were searched in the NCBI non-redundant database, identifying an additional 1 % of the CDSs. In total, about 17,000 individual protein coding genes (Arctic fox1 = 17,136, Arctic fox2 = 17,483 and red fox = 17,310) that are unique in the database could be identified for each of the three fox samples after annotation.

### Identification of 57 genes under positive selection in the two fox species

We identified a total of 11,694 orthologous genes between Arctic and red fox, of which 5,138 remained after the different quality filtration steps for further analyses. Of these, 57 candidate genes were identified to be under positive selection with ω > 1. Functional annotation using Biomart against the dog genome and non-redundant database identified 36 genes to be associated with various important biological and molecular functions (Additional file 4: Table S3). The pairwise mean ω value for all the orthologous genes was 0.057 and a histogram of all the pairwise ω values shows the overall rate of selection on the orthologous genes, implying strong negative selection for most of the genes (Additional file 5: Figure S2). Out of 57 only 36 have been annotated, the rest of the other genes that could not be annotated with Biomart or non-redundant database are therefore most likely fox-specific transcripts. In addition to the pairwise selection analyses, a branch site test was performed for the Arctic fox and red fox branch, respectively. An orthology search identified 5,860 orthologs among four carnivores (Arctic foxes, red fox, dog and panda) of which 4,937 genes remained for the branch site test after the removal of all gaps. The branch site test identified four genes to be under positive selection in the Arctic fox transcriptome and eight genes in the red fox transcriptome. Only two genes (*RNASEH2C* and *TK2*) were identified to be under positive selection by both methods, pairwise and branch-site analyses. The reason for this limited overlap may be a consequence of different methodologies and dataset sizes. The ten candidate genes additionally identified by the branch site test complement the findings by the pairwise analyses. The annotation indicates that the identified positively selected genes of the branch site test are involved in molecular and cellular functions such as lipid metabolism and nuclear chromatin fragmentation (Table 2).

**Table 2:** Positively selected genes with functions, identified in Arctic and red fox using the branch site test

Dog Ensemble transcript ID	Gene Name		Uniprot function	Function (Additional details)
Arctic fox				
ENSCAFT00000048935	*GLTPD1*	Glycolipid transfer protein domain containing 1	Glycolipid binding, Phospholipid binding.	Lipid deposition in chicken [23].
ENSCAFT00000008666	*AKT2*	V-akt murine thymoma viral oncogene homolog 2	ATP binding.	Regulation of fatty acid beta-oxidation and insulin signalling [25, 79].
			Kinase activity.	
ENSCAFT00000001161	*N.A.*	N.A.	Transmembrane transport.	N.A.
ENSCAFT00000021080	*RNASEH2C*	Ribonuclease H2, subunit C	RNA catabolic process.	Involved in Aicardi-Goutieres syndrome [80].
Red fox				
ENSCAFT00000027282	*MAGEE2*	Melanoma antigen family E, 2	N.A.	Member of the melanoma antigen family, unknown function [81].
ENSCAFT00000010371	*APBB1*	Amyloid beta (A4) precursor protein-binding, family B, member 1 (Fe65)	Beta-amyloid binding.	Interacts with the Alzheimer’s disease amyloid precursor protein (APP) [82, 83].
			Chromatin binding.	
ENSCAFT00000006986	*CCDC61*	Coiled-coil domain containing 61	N.A.	N.A.
ENSCAFT00000012394	*LIX1*	Limb Expression 1	N.A	Expressed during chicken and mouse limb development [84, 85].
ENSCAFT00000016648	*SLC6A19*	Solute carrier family 6, member 19	Neurotransmitter: sodium symporter activity.	Involved in Hartnup disorder [86].
ENSCAFT00000032520	*TK2*	Thymidine kinase 2	ATP binding. Thymidine kinase activity.	Required for mitochondrial DNA synthesis. Associated with a myopathic form of mitochondrial DNA depletion syndrome [87].
ENSCAFT00000013454	*EDIL3*	EGF-like repeats and discoidin I-like domains 3	Calcium ion binding. Integrin binding.	Angiogenesis [88, 89] Involved in innate immunity [90].
ENSCAFT00000031005	*DFFB*	DNA fragmentation factor, 40 kDa, beta polypeptide	DNA binding.	Caspase-activated DNAse. Involved in apoptosis [91, 92].
			Desoxyribonuclease activity.	

Gene ontology functions were searched in the Uniprot ontology annotation (Uniprot-GOA)

### Phylogenomic analyses reveal that Arctic fox and red fox diverged 3 million years ago

**Fig. 3:**
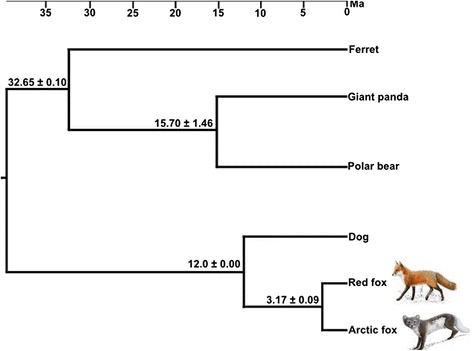
Phylogenetic relationships and divergence times among six carnivore species. The topology was calculated from a 7,589,724 nt long data set, and the divergence times were based on three well established fossil calibration points inside Carnivora. The cat was used as an outgroup (not shown). The values at each branch indicate the estimated mean divergence time (million years ago) ± standard deviation. The scale bar is in million years (Ma)

After screening the carnivore genomes for orthologous genes we retained 6,567 genes with the total length of 7,589,724 nt (2,529,908 amino acids (aa)). The GTR + G model was identified as the most likely substitution model for the nt data and MIX + G was the preferred model for the aa data. Both the nt and aa data sets yielded the same tree topology in maximum likelihood analyses (Fig. 3). Divergence time estimates obtained from using all nt sites and three fossil calibration points indicated that the two fox species diverged 3.17 ± 0.09 Ma (Fig. 3). Divergence time estimation on a data set consisting of only synonymous sites yielded a similar value of 3.16 ± 0.03 Ma.

### Low levels of genetic variation in the Arctic fox

**Fig. 4:**
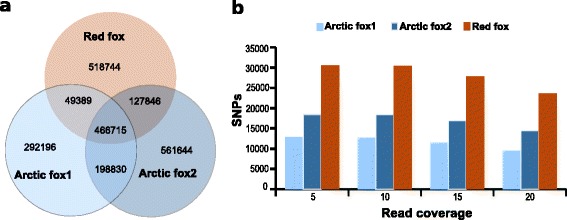
Comparative nucleotide differences (single variable sites) in the three fox individuals. **a** Venn diagram showing the number of variable sites for each of the three fox samples compared to the dog genome, and the degree of shared variable sites among the three foxes. **b** Frequency of heterozygous sites (intra-individual SNPs) in the three fox individuals at different read coverages (5,10,15 and 20)

In total, approximately 70 % of the correctly paired reads from each of the three fox individuals could be mapped to the dog genome. Between the dog and each of the three fox individuals, in total 1,007,130 (Arctic fox1), 1,355,055 (Arctic fox2), and 1,162,694 (red fox) substitutions were found, respectively (Fig. 4a). Approximately 40 % of all substitutions found between the dog genome and Arctic and red foxes were shared between the three fox individuals (Fig. 4a). A search for heterozygous sites (intra-individual SNPs) within each fox individual identified fewer SNPs in the two Arctic foxes as compared to the red fox. Increasing the threshold for the minimal read coverage increased the absolute values somewhat, but not the relative ones (Fig. 4b). The SNPs identified within the three fox individuals will provide a valuable resource for population genetic studies.

Five genes stretching exon and intron boundaries (3.1 kbp) were selected for Sanger-sequencing to verify the quality of the assembled transcriptome data. Amplification and sequencing of the same fox individuals that were used for the transcriptome sequencing identified no differences between the Sanger and Illumina data confirming the high quality of *de novo* assemblies. In total, 17 non-synonymous and four synonymous substitutions were identified among the three individuals. Amplification and sequencing of three additional fox individuals (one Arctic fox and two red foxes) revealed 22 additional substitutions (Additional file 6: Table S4). Nucleotide and haplotype diversity levels were similar or higher in red foxes compared to Arctic foxes (Additional file 6: Table S4). Genetic variation networks (Additional file 7: Figure S3) show that for theses five genes, haplotypes were not shared between red and Arctic foxes.

### Numerous microsatellites suitable for population studies identified in the transcriptome

A total of 952 and 969 di-, tri- and tetra-repeat unit microsatellites were identified in the Arctic and red fox transcripts respectively. While the sequences have not been tested for variability in population samples from the two species yet, they were selected for numerous repeat units. Thus, the identified microsatellite loci will be a valuable resource for future population studies (Additional file 8: Table S5). Trinucleotide microsatellites represented 60 % of the total number of identified microsatellites, Additional file 9: Table S6 lists newly designed 296 microsatellite primer pairs each for Arctic and red foxes that can be used for population genetic studies.

## Discussion

The present study identified positively selected genes in the transcriptomes of the red and the Arctic fox, two species that thrive in different climatic conditions. Arctic and red foxes evolved different morphological, physiological and behavioral adaptations [2, 4]. Pairwise positive selection analyses identified positive selection signatures in 57 of the protein-coding genes of which 36 genes could be successfully annotated and branch site analyses identified four and eight genes in the Arctic and red fox respectively, to be under positive selection. We strived for reduced potential false signals of positive selection introduced during the *de novo* assembly by selecting transcripts of high abundance [19, 20] and *in silico* validating each CDS in two individuals.

Sanger sequencing of five randomly selected genes found no differences between the Illumina and the Sanger sequenced genes, experimentally verifying the stringent data generation approach. Validation of CDS is crucial in positive selection analyses, as false signals can arise from sequencing and assembly artifacts and can skew the results and the interpretation of the data [21].

### Fatty acid metabolism genes are under positive selection in the Arctic fox

We found that 1 % of the genes appear to be under positive selection (ω > 1), a value that is comparable to transcriptome studies in other mammals [22] and indicates widespread negative selection or neutrality at protein-coding sequences. Genes that are expected to be under positive selection had various important molecular and biological functions (Additional file 4: Table S3) including energy metabolism (*DDX20, TK2*), cardiac gene regulation (*SRFBP1*), apoptosis (*TIA1*) and blood coagulation (*GGCX*).

Specifically, the branch site test for positive selection identified two genes related with fatty-acid metabolism to be under positive selection in the Arctic fox: a) the Glycolipid transfer protein domain containing 1 (*GLTPD1*) [23] and b) V-akt murine thymoma viral oncogene homolog 2 (*AKT2*) [24]. The *AKT2* is also involved in insulin signaling in adipose cells by binding to vesicles containing *GLUT4* (glucose transporter) [25]. Insulin plays a central role in the regulation of several metabolic processes, and is involved in glucose uptake into muscle and fat tissue, stimulation of lipogenesis, glycogen and protein synthesis, and inhibition of lipolysis, glycogenolysis and protein breakdown [26]. Arctic foxes experience seasonal and yearly fluctuations in their body fat composition, and need to respond to starvation periods during winter and react to extremely low temperatures by thermoregulation [27]. In addition Arctic foxes have high-energy demands during the reproductive season in spring. While red foxes also have to cope with seasonal fluctuations in food availability, this fluctuation is less pronounced [28]. Hence, genes involved in fatty-acid and glucose metabolism such as *GLPTD1* and *AKT2* may play a role in adaptation to strong seasonal fluctuations as experienced by Arctic foxes. It is noteworthy that fatty-acid metabolism pathways have been identified to be under positive selection in a recent study on polar and brown bear genomes [11]. Energy demands and food availability also vary in polar bears and they seem to have similar evolutionary constraints on fat metabolism compared to Arctic foxes [29]. Thus the metabolism of polar bears and Arctic foxes appears to be similarly affected by adaptive pressures to a life under arctic conditions and consequently selection pressure affects similar metabolic pathways.

### Arctic and red foxes diverged during the late Pliocene at 3.17 Ma

The three fox transcriptomes yielded a large number of protein-coding genes that could be used for evolutionary studies. About 17,000 different protein-coding genes were identified by comparisons to the dog genome from each fox individual by pooling cDNA from three different tissues. This approach yielded 3,000 more genes per transcriptome than a previous study, based only on brain tissue [30]. Thus, relatively complete fox transcriptomes, in this case representing 68 % of the annotated 25,160 genes of the dog genome, can economically be produced for non-model organisms.

The CDS based time estimate for the divergence of the red and Arctic fox from a common ancestor at 3.17 +/− 0.09 Ma is slightly older than a previous estimate based on 22 nuclear loci and 3 mitochondrial (mt) genes and has a much narrower confidence range [3]. The sister species to the Arctic fox are the kit (*Vulpes macrotis*) and the swift fox (*Vulpes velox*), two desert-adapted species occurring in North America [3, 31, 32], while the red fox is more distantly related. Perini et al. [3] reported a divergence time estimate of the Arctic and the kit fox of about 0.9 Ma. Thus, given the congruence of the divergence time estimates from our phylogenomic analysis and from Perini et al. [3], and assuming that the Arctic fox diverged from a southern distributed, swift/kit fox-like ancestor [31] the adaptation of the Arctic fox to the arctic environment most likely began at around 0.9 Ma. Further, the divergence time of the red fox from the corsac fox (*Vulpes corsac*), a closely related species that is adapted to arid conditions [33], was estimated at about 1.8 Ma [3]. Both divergences thus occurred in a time frame during which global temperatures declined and fluctuated between warm and cold phases, providing the environmental setting for the evolution of many species pairs with differential ecological adaptations. Indeed, divergence time estimates between polar bears and brown bears at about 0.34-0.93 Ma [11, 34] coincide with the divergence time estimates of the Arctic and the red fox from their respective arid-adapted sister species.

### The red fox is genetically more variable than the Arctic fox

Compared to the red fox, fewer heterozygous sites (intra-individual SNPs) were found in the transcriptome of the Arctic fox, a finding that was verified by Sanger sequencing in additional Arctic and red fox individuals. This is in agreement with previous studies that have identified a lower level of nucleotide diversity for mt DNA of Arctic foxes [35] compared to red foxes [36]. This is probably a consequence of differences in their distribution ranges and population sizes and of bottlenecks in the Arctic fox population during unfavorable climatic periods [35]. Similar observations have been made in polar bears, which have lower levels of nucleotide diversity for mt DNA and nuclear DNA than brown bears [11, 34]. A lower genetic variation in arctic specialists compared to temperate-zone adapted generalists is expected [37] because during Pleistocene warm phases and during the Holocene, the distribution ranges of arctic specialists declined, resulting in genetic bottlenecks. Although temperate-zone adapted species also suffered from bottlenecks during Pleistocene cold phases, generalists like red foxes and brown bears were able to disperse southwards and to quickly re-colonize suitable habitats after deglaciation [36, 38].

## Conclusions

Transcriptome sequencing is an economic strategy to assemble a large number of protein-coding genes in non-model organisms for which genome sequences are not yet available. This first comparative transcriptome study of the Arctic and the red fox revealed a number of candidate genes that are possibly involved in adaptations to different climatic conditions. Fatty acid metabolism genes are under positive selection in the Arctic fox similar to the polar bear and seem generally to be crucial for mammalian survival under arctic conditions. The SNPs and a large number of microsatellites markers discovered in the fox transcriptome sequences will provide a rich resource to establish new markers for future population genetic studies of nuclear genes that can complement mt DNA based studies [35, 36, 39].

## Methods

### RNA isolation and transcriptome sequencing

Brain, liver, and kidney tissue were harvested from two Arctic foxes (*Vulpes lagopus*) and one red fox (*Vulpes vulpes*) within less than 24 h post-mortem and were stored in RNA later (Ambion) (Additional file 10: Table S7). The red fox sample came from Kronoby, Finland and was legally obtained during hunting season. The Arctic fox samples originated from captive individuals. No ethical approval or permit for animal experimentation were required as the animals were not killed specifically for this study.

Total RNA was isolated from 100 mg of brain, liver, and kidney tissue using the acid guanidinium thiocyanate (GTC)–phenol–chloroform extraction method [40]. One ml freshly prepared Sol-D solution [GTC solution (4 M guanidinium thiocyanate, 0.5 % N-laurylsarcosine, 25 mM sodium citrate (pH 7.0), 0.1 M β-mercaptoethanol] was added to 100 mg homogenized tissue. RNA extracts were purified by subsequent acid GTC–phenol–chloroform extraction. RNA quality and integrity were evaluated by measuring 260–230 nm and 260–280 nm absorbance ratios (Implen NanoPhotometer™) and by denaturing formaldehyde–agarose gel electrophoresis [41]. Per extraction, 24–270 μg total RNA were recovered. Extracts from each of the three tissues were combined into one pool per individual containing 100 μg of total RNA.

Poly-adenylated RNA was captured using Oligo-dT magnetic beads (mRNA purification beads, Dynal). cDNA was generated using first and second-strand synthesis using the SuperScript II reverse transcriptase (Invitrogen) and a nested-poly-T oligonucleotide. A specific oligonucleotide was ligated to the 5′ end to allow for amplification as described by [42]. Normalization was performed as described by [42], using DSN, a double-strand specific nuclease (Evrogen). The normalized cDNA was randomly sheared by a Biorupter (Diagenode) into 100–400 bp fragments and sequenced on a HiSeq2000 (Illumina) at GenXPro GmbH (Frankfurt, Germany).

### Read cleaning and *de novo* assembly of transcriptomes

Raw sequence reads were cleaned for RNA adapters using Tagdust [43]. Sickle [44] trimmed the cleaned reads for low quality bases (Q < 20) with minimum read length of 30 bases. Trinity [45] assembled the cleaned reads using default parameters and a minimum contig length setting of 200 nt. *De novo* assembly was performed separately for the three samples (two Arctic fox and one red fox samples) as well as for a pool of reads from both Arctic foxes. Large amounts of misassembled transcripts/contigs, erroneous and poorly supported contigs can arise during the assembly. Therefore, all reads were mapped back to their respective assembled transcripts using Bowtie2 [46]. Abundance estimation in terms of FPKM value was calculated using eXpress [47] and transcripts ≥ 1 FPKM were retained. Next, clustering of contigs was done using CD-HIT-EST with 95 % similarity [48] to further cluster the similar contigs together.

### Functional annotation of the transcripts and coding sequences

Sma3s program was used to search the transcripts [49] resulting from the *de novo* assembly in the Uniprot database to obtain associated GO terms (ftp://ftp.ebi.ac.uk/pub/databases/uniprot/). Blast2GO assembled the GO terms into three functional GO categories, associated with biological processes, molecular functions and cellular components based on the annotation file from Sma3s [50]. To reduce computational time GOslim implemented in Blast2GO was used with ontology scores of at least two. Transdecoder implemented in Trinity [45] predicted CDS from the filtered transcripts. All predicted fox CDS were further searched using BLAST [51] in the well annotated human protein database from Ensemble and the non-redundant uniprot database using an e-value of 1e-5.

### Orthology identification and selection of correct coding sequences

To identify only those orthologous genes present in all three fox individuals Proteinortho was used with all genes of the species in each orthologous group with algebraic connectivity of one [52]. The dataset of orthologous Arctic and red fox CDS were further screened to identify and remove CDS that were suspected to have sequencing and assembly artifacts, as this is known to influence positive selection analysis. The two orthologous Arctic fox CDS were pairwise aligned using EMBOSS Needle [53] with the parameters “-gapopen 10 -gapextend 0.5”. Only genes that were 100 % identical in the two Arctic fox individuals were kept for further analysis, treating genes with large distance values as a result from potential artifacts. As only one red fox individual was available, the published transcriptome from a silver fox (*Vulpes vulpes),* which are red foxes characterized by a coat-color variant [30], was downloaded from the Short Read Archive (SRA) (SRA029285.1). The silver fox reads were mapped against the red fox CDS using BWA [54] and consensus sequences were generated using the samtools [55]. The silver fox and red fox CDS were pair-wise aligned and only genes that were 100 % identical were kept for the final pairwise positive selection analysis. This approach, with verification in two independently sequenced individuals for each species, minimized assembly artifacts to ensure that only well assembled protein-coding sequences were included into subsequent analyses.

### Identification of positively selected genes in foxes using pairwise and branch site selection tests

The identified orthologous Arctic and red fox CDS were pairwise aligned and used for pairwise positive selection analysis to identify genes with an ω > 1. All CDS with dS = 0 were discarded. For the pairwise positive selection analysis, first all CDS sequences were translated and aligned using PRANK, which was shown to perform better compared to other sequence aligners used in positive selection analyses [56, 57]. Next the sequences were back-translated to obtain the nt coding sequence alignment using a perl script (Additional file 11). All alignment gaps were removed maintaining the correct reading frame. All alignments shorter than 60 aa were discarded from future analyses. PAML package version 4.4 [9] was used to calculate ω for the pairwise alignments using the yn00 model [6]. For fox transcriptome sequences with an ω > 1, the dog ortholog was identified in the Ensemble dog database and non-redundant database with the blastx [51] using an e-value 1e-5 as threshold to assign their corresponding functions.

In addition to the pairwise selection analysis, a branch site test was performed to identify positive selection on the branches leading to the Arctic and red fox including two additional carnivorous species, dog (*Canis lupus familiaris*) and giant panda (*Ailuropoda melanoleuca*) from Ensembl (http://www.ensembl.org/info/data/ftp/index.html). Similar to the orthology screen in foxes, one-to-one orthologous genes present in all investigated carnivores were determined using Proteinortho [52]. Orthologous aa sequences were aligned using PRANK [56] and were then back-translated to obtain the nt coding sequence alignment. All gaps were removed from the alignments and sequences shorter than 60 aa were discarded. The codeml module implemented in the PAML package [9] was used to perform the branch site test. Alternate (model = 2, NSsite = 2, fix_omega = 1) and null hypothesis (mode l = 2, NSsite = 2, fix_omega = 0 and omega = 1) likelihood values were tested using a chi-square distribution and significance level of 5 %. Only alignments including Bayesian empirical bayes (BEB) sites > 95 % and having a dS < 1 were retained as a positive selected genes. Gene ontology functions were searched in the Uniprot gene ontology annotation (Uniprot-GOA).

### Phylogenomic analyses and divergence time estimates of Carnivora

We took the advantage of several available sequenced genomes to reconstruct the evolutionary history of Carnivora using a phylogenomic data set. The taxon sampling included the Arctic, red fox and the polar bear (*Ursus maritimus*) (http://gigadb.org). Utilizing the two Arctic fox CDS datasets, we generated a common Arctic fox CDS dataset by first identifying CDS present in both individuals using BLAT [58] and combining this with the CDS that were unique for each individual to maximize the amount of CDS.

Other carnivorous genomes were downloaded from the Ensemble database including giant panda (*Ailuropoda melanoleuca,* AilMel 1.0), dog (*Canis lupus familiaris*, canFam3), ferret (*Mustela putorius furo*, musFur1), and cat (*Felis catus*, felCat5). Orthologous genes in the seven species were identified using Proteinortho [52]. All sequence alignments that included at least six species were selected. Protein sequences were aligned using Mafft [59]. The protein alignments were back-translated into nt sequences using a perl script and all gaps were removed. Two datasets were constructed from concatenated alignments based on either aa and nt data. The optimal evolutionary model was identified in Treefinder [60] and was used in all subsequent analyses to reconstruct maximum-likelihood trees for aa and nt data. Three fossil calibration points were used to estimate divergence times using Treefinder [60]: 1) the divergence between Feliformia and Caniformia at 39.7 - 63.8 Ma [61]; 2) the divergence between Ailuropodinae and Ursidae at 11.6 - 39.7 Ma [62]; and 3) the *Canini* and *Vulpini* divergence at 12 Ma [63, 64]. Divergence time estimates were based on the NPRS-LOG method implemented in Treefinder [60]. Mean values and standard deviations were calculated based on branch lengths, for which 1,000 times maximum likelihood bootstraps were obtained. Additionally divergence time was calculated based on the synonymous sites (Ks) from the same data set, using the Seaview Neighbour joining method (version 4) [65] bootstrapped 1,000 times using Phylip, version 3.6 [66].

### Sequence differences among the foxes and dog

The dog genome (canFam3) represents the evolutionarily closest available genome dataset compared to Arctic and red foxes and was therefore retrieved from the UCSC genome database. Single variable sites were obtained by mapping the reads from each of the three fox individuals to the dog genome using the STAR alignment tool including the 2-pass STAR method [67, 68]. GATK haplotype caller was used to call the variable sites from the genome-mapped alignments [69]. The filtering steps included duplicate marking, split ‘N’ Trim and reassigning mapping qualities. Then variant filtration was performed using the parameters “-window 35, −cluster 3, −filterName FS, −filter “FS > 30.0” -filterName QD, −filter QD < 2.0”. VCFtools_0.1.12b [70] was used to obtain shared single nt changes from the vcf files.

To identify heterozygous sites (SNPs) for each fox individual, reads were mapped back to their corresponding assembled transcripts using BWA with default parameters [54]. Samtools was used to remove duplicate reads, which may arise during Polymerase-chain reaction (PCR), and indel alignment score improvement was done to improve the mapping quality [55]. Varscan was used to call the SNPs with parameters of “min-avg-qual 25, p-value 0.01, minvarfreq 0.2 and min-coverage of 5, 10, 15 and 20" [71].

### Evaluation of a subset of genes for assembly artifacts and SNP validation

Total DNA was isolated from muscle samples from red and Arctic foxes (Additional file 10: Table S7) using a standard salt extraction protocol [72]. Primers were designed for five randomly chosen genes for coding regions from the Arctic fox, the red fox and including dog genome sequences (Additional file 12: Table S8) [73]. PCR was performed using 15 ng of genomic DNA, and each PCR setup contained no-template controls (Additional file 10: Table S7). Standard agarose gel electrophoresis was used to detect PCR products, which were cycle sequenced with BigDye 3.1 chemistry (Applied Biosystems, Foster City, CA, USA) in both directions according to the manufacturer’s recommendations, and detected on an ABI 3100 instrument (Applied Biosystems). Electropherograms were checked manually, and the obtained sequences were aligned using the ClustalW function in Geneious 5.6.6 [74] (Biomatters, Auckland, New Zealand).

Haplotypes were deduced using PHASE implemented in the software DnaSP v5.0 [75], allowing for recombination within haplotypes. Sequence diversity and differentiation statistics for three red and three Arctic foxes were calculated in DnaSP. For reconstructing statistical parsimony networks in TCS 1.21 [76], indels were treated as single mutational events, and gaps as a fifth character state. Longer gaps were treated as single mutational changes and the connection probability limit was set to 0.95.

In addition, we also checked the quality and expression patterns in terms of FPKM for orthologous genes. Reads of one species were mapped using Bowtie2 [46] and FPKM was calculated using eXpress [47] to their own assembly and also to the other species assembly. Later using R correlation plots were made for all the orthologous genes in both the species.

### Identification of microsatellites in the transcriptome data

The Arctic fox assembly, which was obtained from a pooled assembly of two individuals and red fox transcripts were screened for microsatellites using the program SciRoKo [77]. The minimum number of microsatellite repeats was set to seven for dinucleotides and to five for trinucleotides and tetranucleotides, to increase the likelihood, that they are variable. Primers were designed using the software Primer 3.0 [78] setting the parameters to a primer product size range of 100–250 bp, primer optimum size of 20 bp, and an optimum melting temperature (Tm) = 60.0 °C.

### Availability of supporting data

The raw reads are available at the European nucleotide archive (ENA) database. Study accession number: PRJEB7790. The Sanger sequences are available at ENA accession numbers LN680791-LN680850.

## Additional files

Additional file 1: Table S1. Summary of the reads from the transcriptome after adapter removal and quality trimming. Lab ID is shown in brackets. Additional file 2: Table S2. Details of the *de novo* assembled contigs/transcripts based on the abundance estimation (FPKM) and clustering. Lab ID is shown in brackets. Additional file 3: Figure S1. Comparative correlation plots of FPKM for the orthologous genes in different fox assemblies. A : Correlation plot of AF1 with RF. B: Correlation plot of AF2 with RF. C: Correlation plot of RF with AF1. D:Correlation plot of RF with AF2. Note (AF1corresponds to Arctic fox1, AF2 to Arctic fox2 and RF to Red fox). Additional file 4: Table S3. Candidate genes identified to be under positive selection using pair-wise positive selection analysis. The gene functions are according to the Ensembl database. Additional file 5: Figure S2. Histogram showing the pairwise ω values of all orthologous genes between the Arctic and red fox. Additional file 6: Table S4. Summary statistics of five genes that were validated through Sanger sequencing in arctic Arctic and red foxes. N number of individuals; H number of haplotypes; S number of segregating sites; syn. number of synonymous sites; non-syn. number of non-synonymous sites; Hd haplotype diversity; π nucleotide diversity; D Tajima’s D. Additional file 7: Figure S3. Statistical parsimony networks for five genes in Arctic and red foxes, including exon and intron sequence. Circle areas are proportional to haplotype frequencies and inferred intermediate states are shown as black dots. N = Non-synonymous substitutions; S = synonymous substitutions. Allele color codes: blue = Arctic foxes; orange = red foxes; black = dog. Note that for some loci, dog and fox haplotypes were too divergent to be connected at the 95 % credibility limit. Additional file 8: Table S5. Details of different types of microsatellites identified in the Arctic and red fox transcriptomes. Frequency of di-, tri-, tetra- repeat motifs are shown. Additional file 9: Table S6. Selected microsatellite markers with their corresponding primers. Additional file 10: Table S7. Details of the fox samples and sequences used in the study. Individuals 1, 2 and 4 were used for transcriptome sequencing. Additional file 11:
**Perl script for the backtranslation of amino acid alignments into nucleotide sequence alignments.**
Additional file 12: Table S8. Primers (in 5′ to 3′ orientation) and amplification conditions for five gene fragments selected for Sanger-sequencing verification. The gene name, the exon and intron numbers for the dog genome are given; size for nuclear markers denotes expected amplicon size in base pairs (bp) in dog; T annealing temprature range used in touchdown PCR.
