# Supplementary figures and images for: Genetic signatures of adaptation revealed from transcriptome sequencing of Arctic and red foxes
Source: BMC Genomics. 2015 Aug 7;16(1):585. doi: 10.1186/s12864-015-1724-9 (PMC4528681; doi:10.1186/s12864-015-1724-9)

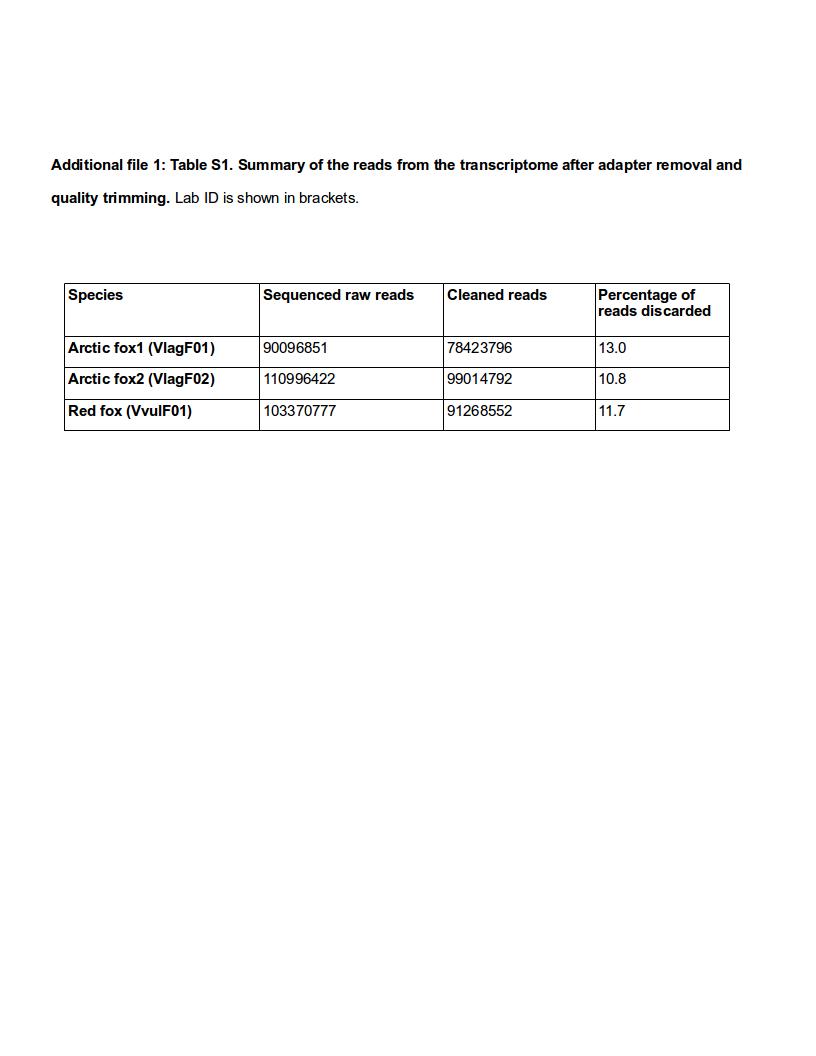

Supplement: Additional file 1: Table S1. — Summary of the reads from the transcriptome after adapter removal and quality trimming. Lab ID is shown in brackets. [file 12864_2015_1724_MOESM1_ESM.png]

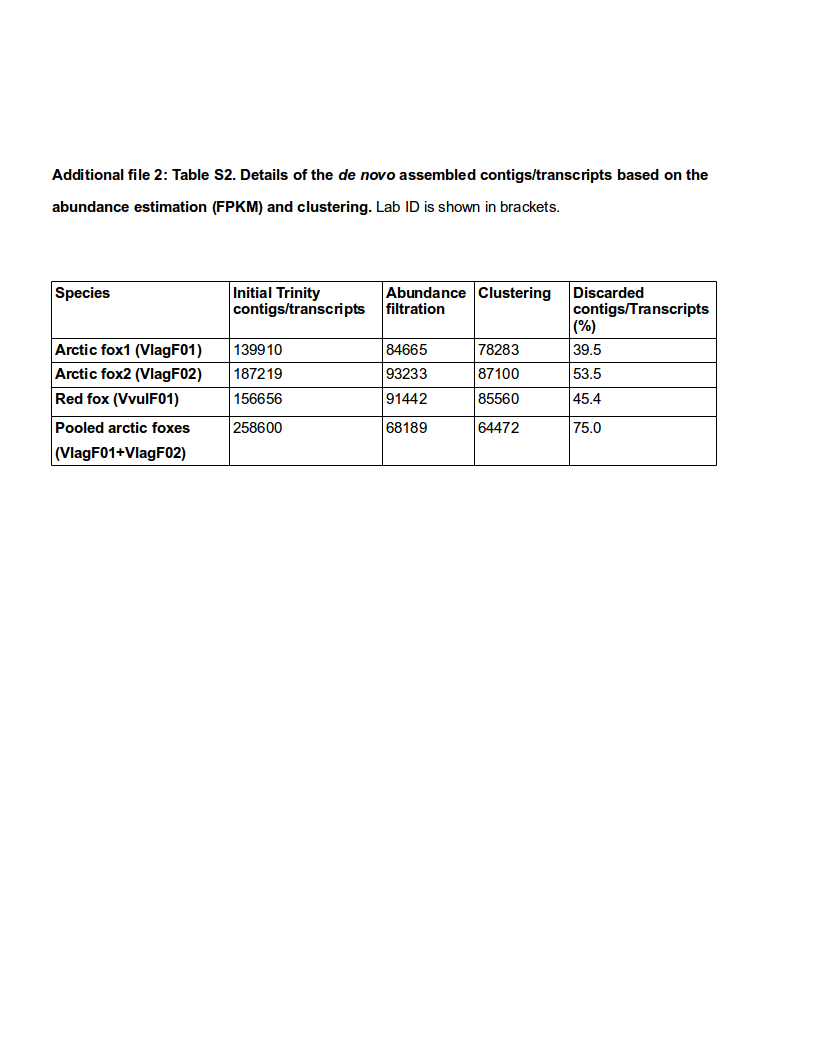

Supplement: Additional file 2: Table S2. — Details of the de novo assembled contigs/transcripts based on the abundance estimation (FPKM) and clustering. Lab ID is shown in brackets. [file 12864_2015_1724_MOESM2_ESM.png]

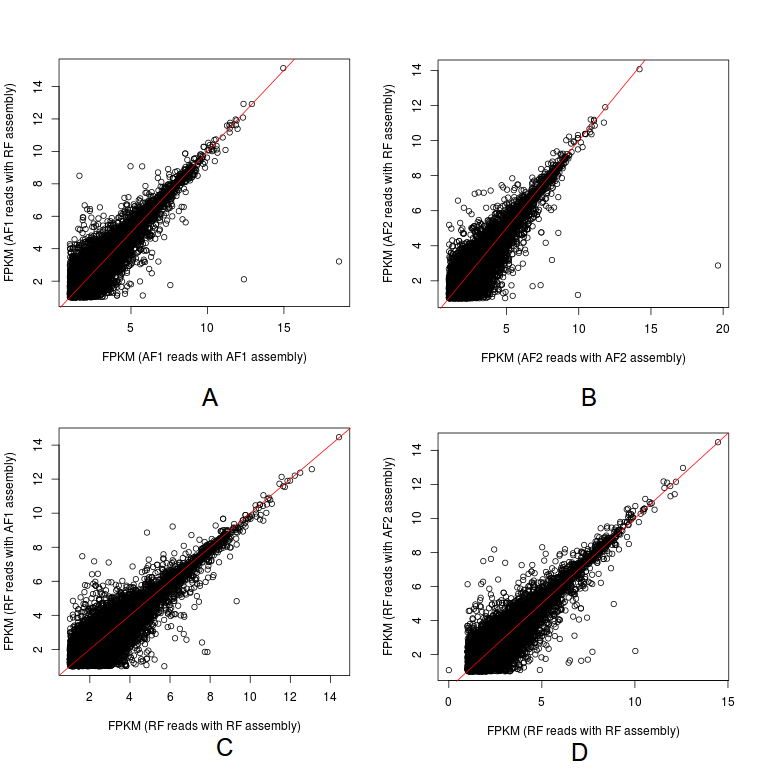

Supplement: Additional file 3: Figure S1. — Comparative correlation plots of FPKM for the orthologous genes in different fox assemblies. A : Correlation plot of AF1 with RF. B: Correlation plot of AF2 with RF. C: Correlation plot of RF with AF1. D:Correlation plot of RF with AF2. Note (AF1corresponds to Arctic fox1, AF2 to Arctic fox2 and RF to Red fox). [file 12864_2015_1724_MOESM3_ESM.png]

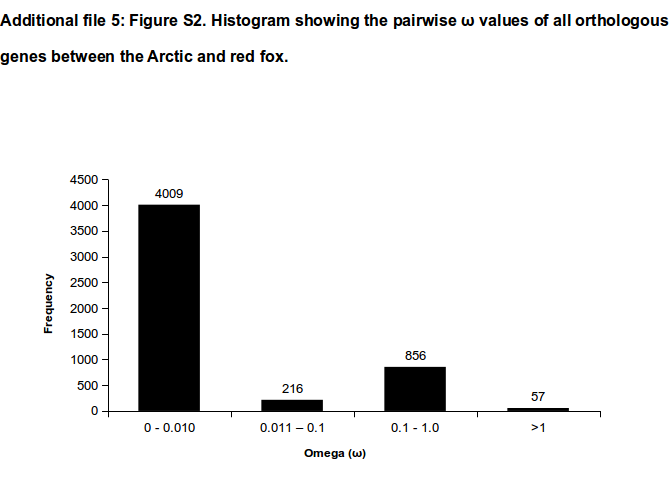

Supplement: Additional file 5: Figure S2. — Histogram showing the pairwise ω values of all orthologous genes between the Arctic and red fox. [file 12864_2015_1724_MOESM5_ESM.png]

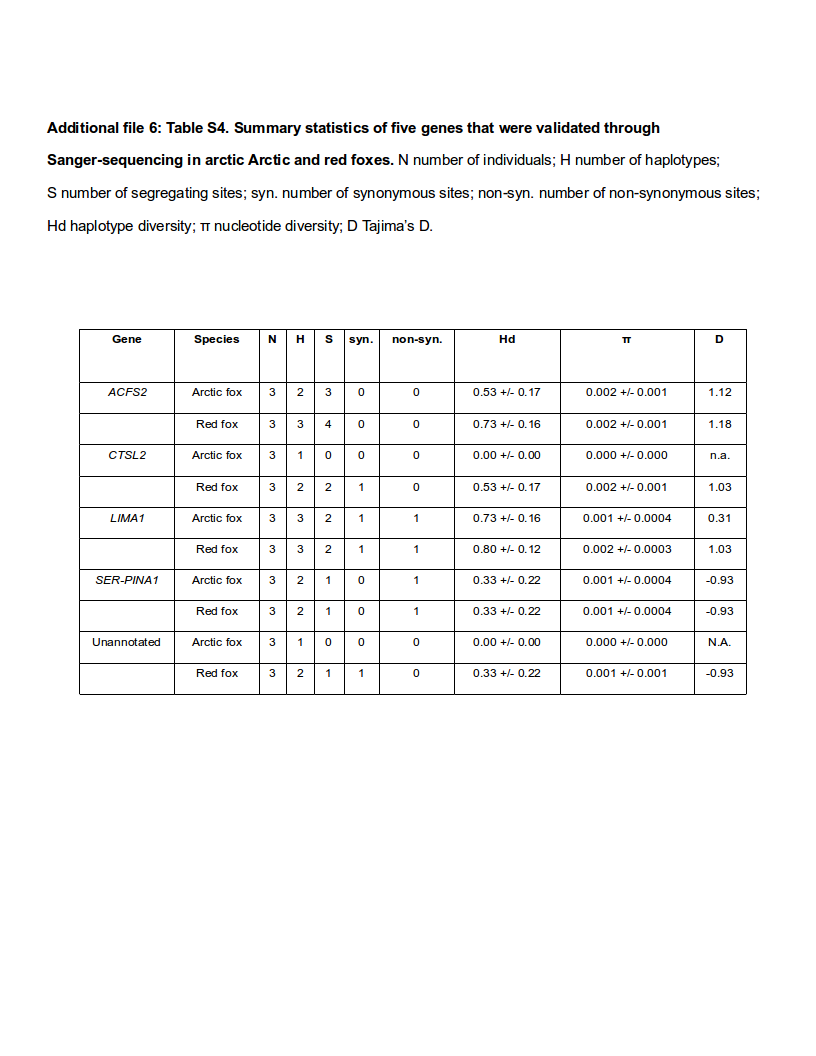

Supplement: Additional file 6: Table S4. — Summary statistics of five genes that were validated through Sanger sequencing in arctic Arctic and red foxes. N number of individuals; H number of haplotypes; S number of segregating sites; syn. number of synonymous sites; non-syn. number of non-synonymous sites; Hd haplotype diversity; π nucleotide diversity; D Tajima’s D. [file 12864_2015_1724_MOESM6_ESM.png]

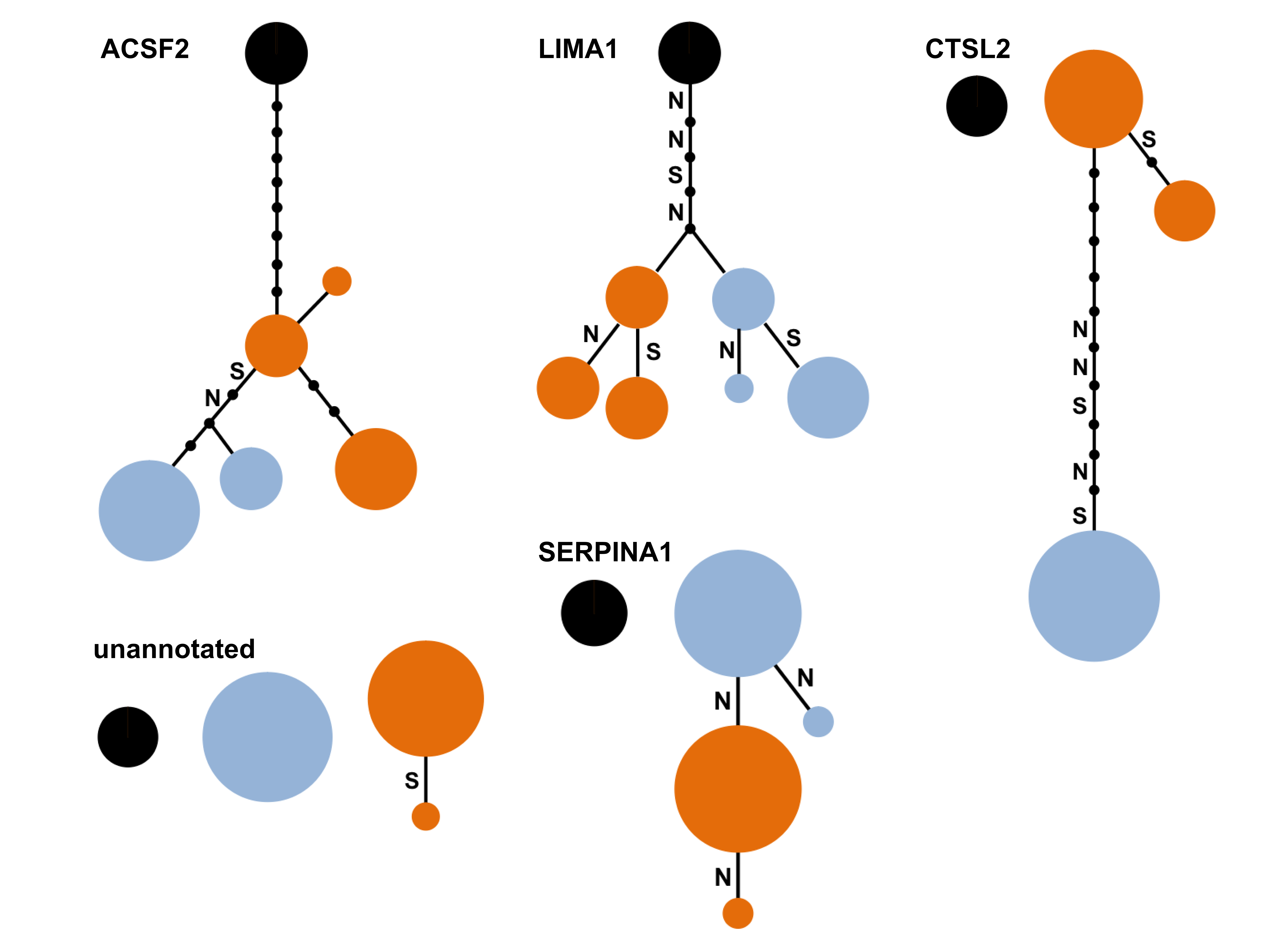

Supplement: Additional file 7: Figure S3. — Statistical parsimony networks for five genes in Arctic and red foxes, including exon and intron sequence. Circle areas are proportional to haplotype frequencies and inferred intermediate states are shown as black dots. N = Non-synonymous substitutions; S = synonymous substitutions. Allele color codes: blue = Arctic foxes; orange = red foxes; black = dog. Note that for some loci, dog and fox haplotypes were too divergent to be connected at the 95 % credibility limit. [file 12864_2015_1724_MOESM7_ESM.png]

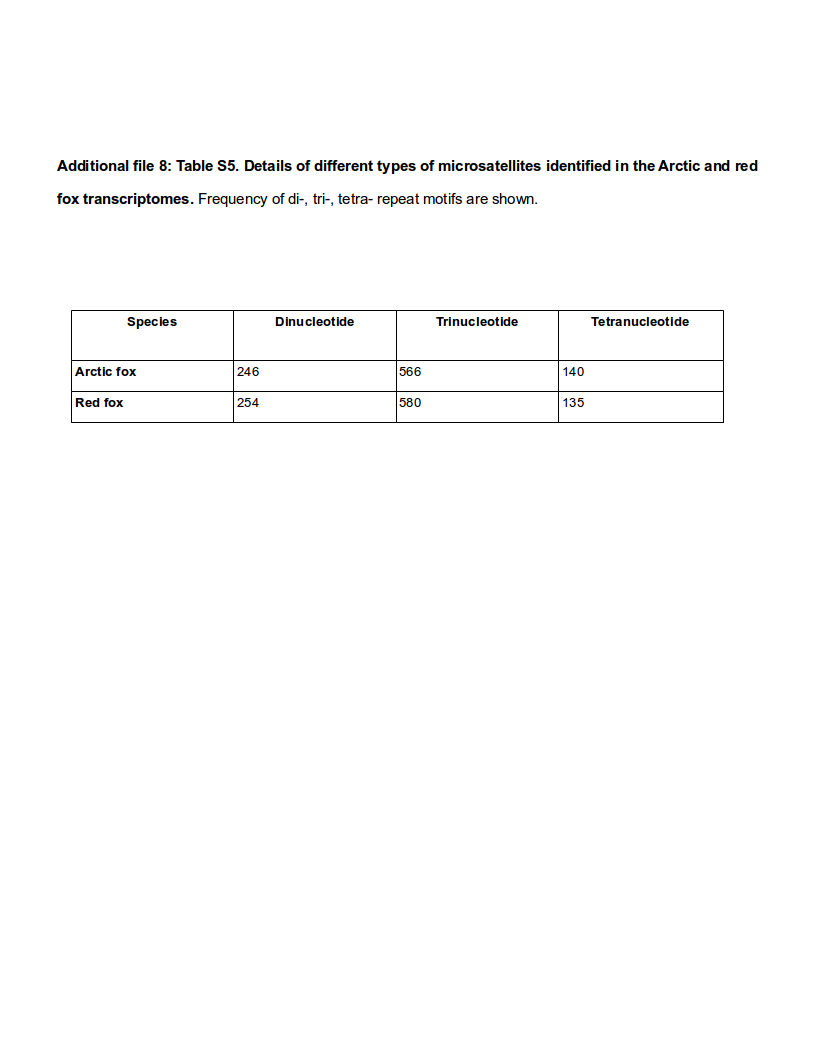

Supplement: Additional file 8: Table S5. — Details of different types of microsatellites identified in the Arctic and red fox transcriptomes. Frequency of di-, tri-, tetra- repeat motifs are shown. [file 12864_2015_1724_MOESM8_ESM.png]

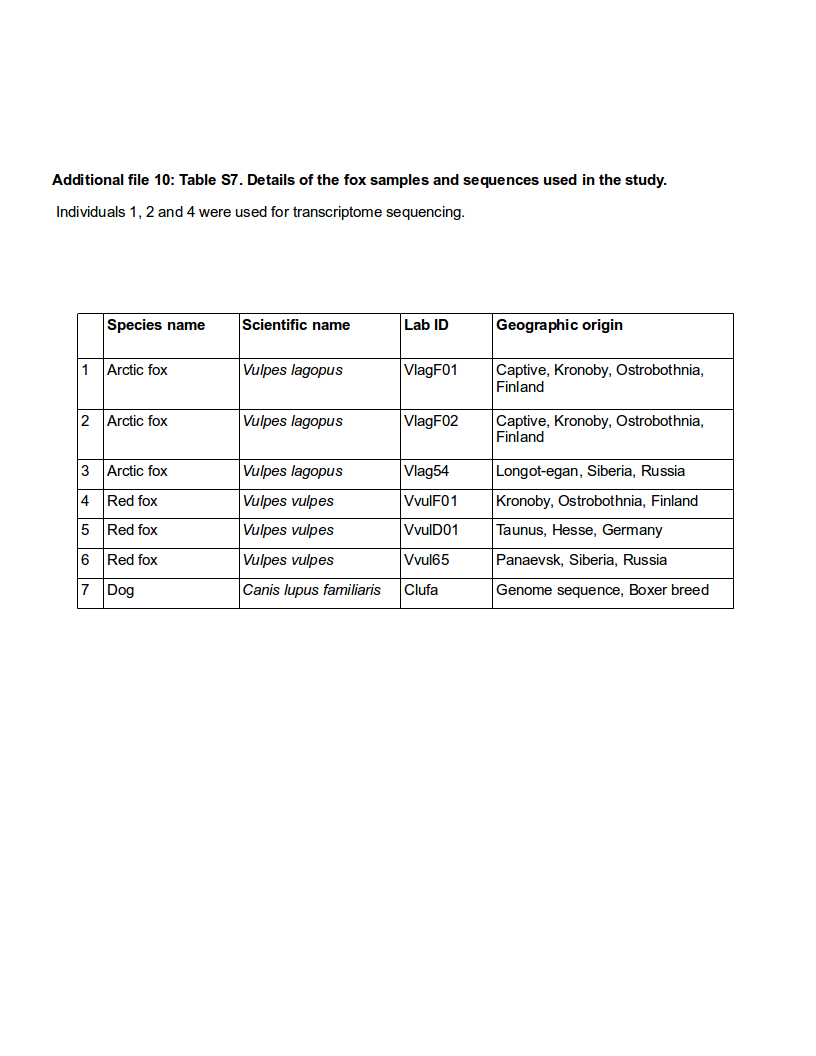

Supplement: Additional file 10: Table S7. — Details of the fox samples and sequences used in the study. Individuals 1, 2 and 4 were used for transcriptome sequencing. [file 12864_2015_1724_MOESM10_ESM.png]

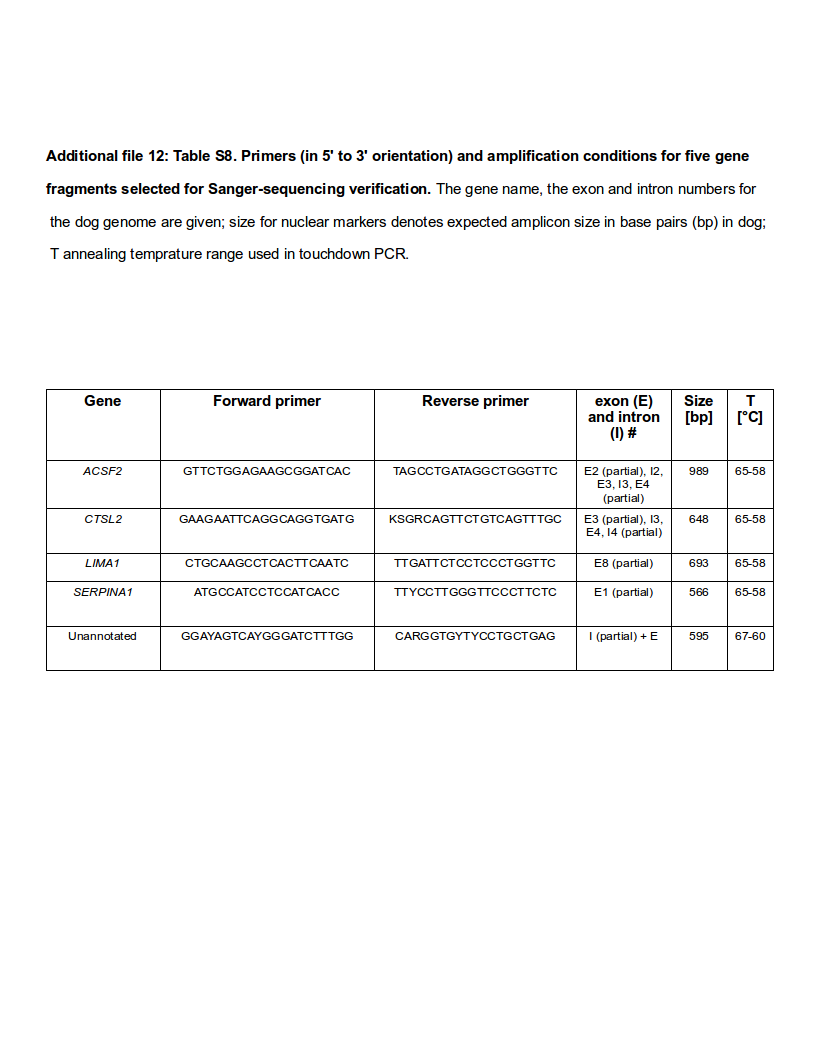

Supplement: Additional file 12: Table S8. — Primers (in 5′ to 3′ orientation) and amplification conditions for five gene fragments selected for Sanger-sequencing verification. The gene name, the exon and intron numbers for the dog genome are given; size for nuclear markers denotes expected amplicon size in base pairs (bp) in dog; T annealing temprature range used in touchdown PCR. [file 12864_2015_1724_MOESM12_ESM.png]
